# Increased sea ice cover alters food web structure in East Antarctica

**DOI:** 10.1038/s41598-019-44605-5

**Published:** 2019-05-30

**Authors:** Loïc N. Michel, Bruno Danis, Philippe Dubois, Marc Eleaume, Jérôme Fournier, Cyril Gallut, Philip Jane, Gilles Lepoint

**Affiliations:** 1 Laboratory of Oceanology, Freshwater and Oceanic Sciences Unit of reSearch (FOCUS), University of Liège (ULg), Liège, Belgium; 2 Marine Biology Laboratory, Université Libre de Bruxelles (ULB), Brussels, Belgium; 3 Institut de Systématique, Évolution, Biodiversité (ISYEB), Muséum National d’Histoire Naturelle, CNRS, Sorbonne Université, EPHE, Paris, France; 4 CNRS, UMR 7208 BOREA, Biological Marine Station, National Museum of Natural History (MNHN), Concarneau, France; 5 Institut de Systématique, Évolution, Biodiversité (ISYEB), Sorbonne Université, CNRS, MNHN, EPHE, Station marine de Concarneau, Concarneau, France; 6 Aquarium de Paris - Cinéaqua, Paris, France; 7 Present Address: Ifremer, Centre de Bretagne, REM/EEP, Laboratoire Environnement Profond, Plouzané, France

**Keywords:** Stable isotope analysis, Ecological networks, Ecosystem ecology, Climate-change ecology

## Abstract

In recent years, sea ice cover along coasts of East Antarctica has tended to increase. To understand ecological implications of these environmental changes, we studied benthic food web structure on the coasts of Adélie Land during an event of unusually high sea ice cover (i.e. two successive austral summers without seasonal breakup). We used integrative trophic markers (stable isotope ratios of carbon, nitrogen and sulfur) to build ecological models and explored feeding habits of macroinvertebrates. In total, 28 taxa spanning most present animal groups and functional guilds were investigated. Our results indicate that the absence of seasonal sea ice breakup deeply influenced benthic food webs. Sympagic algae dominated the diet of many key consumers, and the trophic levels of invertebrates were low, suggesting omnivore consumers did not rely much on predation and/or scavenging. Our results provide insights about how Antarctic benthic consumers, which typically live in an extremely stable environment, might adapt their feeding habits in response to sudden changes in environmental conditions and trophic resource availability. They also show that local and/or global trends of sea ice increase in Antarctica have the potential to cause drastic changes in food web structure, and therefore to impact benthic communities.

## Introduction

Antarctica is one of the most productive and biodiverse regions of the marine realm, and species living there have to cope with extreme, yet stable environmental conditions such as low temperatures or intense seasonality. These narrow environmental limits likely persisted over most of the last million years, and could have been a defining factor for Antarctic fauna’s evolutionary history^1,2^. Environmental stability in Antarctica is jeopardized by anthropogenic climate change that has fast and contrasting impacts on Southern regions^1^. While the western Antarctic Peninsula is one of the most rapidly warming regions in the world, resulting in sea ice cover decrease, the sea ice cover tends to increase in some parts of East Antarctica, possibly in relation with changes in atmospheric circulation^3,4^. These contrasting changes affect both the spatial (i.e. the sea ice concentration at a given location) and the temporal extent (i.e. the duration of the season during which sea ice is present) of the Antarctic sea ice cover^5,6^. Moreover, high inter-annual variability and occurrence of global change-induced extreme events^7^ complicate assessment of these environmental changes and understanding of their effects on biological organisms.

The surroundings of the Dumont-d’Urville research station (Adélie Land, East Antarctica) provide a spectacular example of sea ice increase along the Antarctic littoral. In the Western Pacific Ocean Sector of East Antarctica (90° E–160° E), landfast ice typically starts to form in April and reaches maximum extent in mid- to late September. Seasonal breakup generally occurs in November, and most coastal zones of this sector are free from landfast ice from December to March^8^. Local observations around Dumont-d’Urville station confirm this pattern, although there is inter-annual variability in the period at which the seasonal breakup occurs, which can range from early November to early January^9,10^. Regardless, sea ice season length in Adélie Land tended to increase from 1980 to 2002^11^. Besides those long-term trends, events of extreme sea ice development have been recorded in recent years, culminating in austral summers during which no breakup occurred in the surroundings of Dumont-d’Urville station. While a classic seasonal breakup, corresponding to the situation described above, was observed during all summers from 1980 to 2013, total absence of breakup was noted in 3 of the 5 last years (austral summers 2013–2014, 2014–2015 and 2016–2017), during which islands of the Géologie archipelago remained connected by sea ice throughout summer (French Polar Institute Paul-Emile Victor – IPEV, pers. comm.). These recent trends could be linked with regional features such as calving of the Mertz glacier in 2010, as ice shelves, sea ice and fast ice seem to be bound by complex oceanic processes in coastal Adélie and George V lands^12^.

Impacts of such a dramatic increase in sea ice cover on benthic ecosystems are poorly understood, as ecological effects of sea ice loss have received more attention than effects of sea ice expansion^13^. Sea ice is nevertheless a major environmental driver of marine ecological processes in Antarctica, where food web dynamics are strongly intertwined with sea ice conditions^14,15^. Sea ice exerts direct (through export of sympagic primary production) and indirect (through coupling with oceanographic processes) influence on the nature and abundance of food items available for benthic organisms. Feeding habits of benthic consumers have accordingly been shown to vary between locations featuring different sea ice extent and persistence^14,16^. Moreover, seasonal sea-ice breakups cause strong and highly predictable food pulses of which the benthic organisms are able to take advantage by shifting their dietary niche^17^. Overall, it seems likely that changes in sea ice conditions of the magnitude of those encountered around Dumont-d’Urville station in recent years could influence trophic interactions among Antarctic zoobenthos.

In this context, the main objective of this study was to understand how absence of seasonal breakup could impact the structure of benthic food webs. Marine food webs are complex networks of ecological interactions, but their essential parameters can be summarized using two dimensions, leading to the traditional depiction of food webs as bi-dimensional diagrams^18^. The horizontal dimension of these diagrams encompasses the diversity of producers sustaining the food web. Their vertical structure is dictated by the trophic position of the consumers (e.g. primary consumers, secondary consumers, omnivores, etc.). Here, we sampled biomass-dominant invertebrates around Dumont-d’Urville station, as well as their food items, during two successive austral summers (2013–14 and 2014–15) where no seasonal breakup occurred. We used integrative trophic markers (stable isotope ratios of carbon, nitrogen and sulfur) to build ecological models that were used as proxies of both fundamental food web dimensions. The horizontal dimension was depicted using a mixing model^19,20^ to delineate diets of primary consumers, identify food items supporting benthic communities, and quantify the relative importance of production originating from different compartments (sympagic, pelagic and benthic) for invertebrate nutrition under those peculiar sea ice conditions. The vertical structure of the food web was recreated through a trophic position model^21^ that was used to provide continuous estimates of the distance between benthic consumers and the baseline items supporting them during austral summers without breakup.

## Results

### Stable isotope ratios of food sources and consumers (Table 1)

δ^13^C of food sources covered a wide interval, ranging from −34.6 ± 1.6‰ (*Phyllophora antarctica* blades) to −12.5 ± 1.7‰ (sympagic algae). *Pygoscelis adeliae* guano (δ^13^C = −28.5 ± 0.9‰) and suspended particulate organic matter (δ^13^C = −26.7 ± 0.6‰) also had quite negative δ^13^C. Benthic biofilm had second least δ^13^C of food items (δ^13^C = −20.0 ± 1.4‰). Its value was nevertheless clearly distinct from sympagic algae (Dunn’s post-hoc test, p < 0.0001). *Himantothallus grandifolius* showed inter-organ variation, as blades (δ^13^C = −23.6 ± 1.8‰) were more ^13^C-depleted than stipes (δ^13^C = −21.5 ± 1.1‰; Dunn’s post-hoc test, p = 0.0047) or holdfasts (δ^13^C = −21.9 ± 2.0‰; Dunn’s post-hoc test, p = 0.0203). All consumers δ^13^C were comprised between values of SPOM and sympagic algae, and most mean values were found between −14 and −20‰ (Table 1).

**Table 1:** Sampling details and stable isotope ratios of producers/organic matter pools (top part of the table) and consumers (bottom part of the table).

Higher taxon	Taxon/sample nature	Method	Analysed tissue	N_13–14_	N_14–15_	δ^13^C (‰)	δ^15^N (‰)	δ^34^S (‰)	SIAR
Phaeophyta	*Himantothallus grandifolius*	S	Holdfasts		16	−23.6 ± 1.8	3.3 ± 0.4	17.6 ± 0.5	
	*Himantothallus grandifolius*	S	Stipes		16	−21.5 ± 1.1	3.6 ± 1.3	16.1 ± 1.0	
	*Himantothallus grandifolius*	S	Blades	3	16	−21.9 ± 2.0	9.7 ± 1.2	13.1 ± 3.3	
Rhodophyta	*Phyllophora antarctica*	S	Blades		17	−34.6 ± 1.6	3.4 ± 0.7	16.1 ± 0.9	
-	Sympagic algae	S	Whole material	4	20	−12.5 ± 1.7	5.3 ± 0.5	5.6 ± 2.7	
	Biofilm	S	Whole material	5	22	−20.0 ± 1.4	4.2 ± 0.7	10.5 ± 3.0	
	Suspended particulate organic matter	N	Whole material	3	12	−26.7 ± 0.6	6.4 ± 0.8	18.5 ± 0.8	
	*Pygoscelis adeliae* guano	H	Whole material		21	−28.5 ± 0.9	22.0 ± 1.4	15.9 ± 1.1	
Porifera	Demospongiae Indet.	S	Body fragments		14	−19.2 ± 0.3	8.3 ± 0.5	16.3 ± 0.7	X
	*Hemigellius* sp.	S	Body fragments		19	−20.2 ± 0.5	10.4 ± 1.6	10.9 ± 2.0	
	*Homaxinella balfourensis*	S	Body fragments		22	−19.5 ± 2.2	8.2 ± 1.7	16.2 ± 2.3	X
	*Mycale acerata*	S	Body fragments		17	−20.7 ± 0.4	10.6 ± 2.5	17.0 ± 0.5	
Cnidaria	*Isotealia antarctica*	S	Ectoderm, lower body region		23	−16.2 ± 1.2	10.7 ± 0.8	11.8 ± 1.5	
Nemertea	*Parborlasia corrugatus*	S/T	Body wall, anterior region		24	−19.3 ± 1.6	10.1 ± 1.0	14.8 ± 0.6	
Nematoda	*Deontostoma* sp.	S	30 whole individuals		11	−24.2 ± 1.6	9.3 ± 0.7	14.3 ± 0.7	
Polychaeta	*Flabegraviera mundata*	S	Body wall		22	−15.8 ± 0.7	7.1 ± 1.5	9.5 ± 2.1	X
(Errantia)	*Harmothoe* sp.	S	Whole animal without gut		30	−16.7 ± 1.8	8.7 ± 0.7	13.8 ± 1.6	X
Polychaeta	*Perkinsiana* sp.	S	Whole animal without gut		24	−18.3 ± 1.2	7.5 ± 1.4	14.3 ± 1.8	X
(Sedentaria)	*Polycirrus* sp.	S	Whole animal without gut		19	−16.9 ± 1.0	7.7 ± 1.3	14.7 ± 1.0	X
Sipuncula	*Golfingia* sp.	S	Body wall		14	−19.0 ± 1.2	7.6 ± 0.9	12.7 ± 0.7	X
Pycnogonida	*Ammothea carolinensis*	S	Whole animal without gut		19	−20.8 ± 3.4	11.0 ± 0.9	15.8 ± 0.9	
	*Decolopoda australis*	S	Whole animal without gut		24	−19.9 ± 1.5	9.8 ± 1.1	16.4 ± 1.5	
Amphipoda	*Charcotia obesa*	T	Whole animal without gut		27	−20.6 ± 0.6	9.1 ± 1.3	16.7 ± 0.2	
Bivalvia	*Adamussium colbecki*	S	Shell adductor muscle	3	25	−19.5 ± 0.6	4.5 ± 0.3	14.6 ± 0.9	X
	*Laternula elliptica*	S	Siphon muscle		21	−22.4 ± 1.5	4.4 ± 0.5	15.4 ± 0.6	X
Gastropoda	*Margarella* sp.	S	10 whole animals without shell		11	−18.9 ± 0.5	8.0 ± 0.3	15.8 ± 0.7	X
	*Marseniopsis* sp.	S	Foot muscle		21	−22.8 ± 1.1	8.1 ± 0.1	16.2 ± 0.3	X
	*Trophonella longstaffi*	S	Foot muscle		22	−21.3 ± 0.3	8.0 ± 0.7	16.1 ± 0.1	X
Asteroidea	*Acodontaster* sp.	S	Podial vesicles		11	−18.7 ± 0.7	10.4 ± 0.7	17.3 ± 0.6	
	*Diplasterias brucei*	S/T	Podial vesicles		21	−15.8 ± 1.7	8.6 ± 0.7	16.3 ± 0.8	X
	*Odontaster validus*	S/T	Podial vesicles	5	23	−13.9 ± 0.8	8.7 ± 0.5	15.4 ± 0.9	X
	*Saliasterias brachiata*	S	Whole animal without gut		13	−16.9 ± 0.3	10.2 ± 0.1	16.7 ± 0.4	
Echinoidea	*Sterechinus neumayeri*	S	Aristotle’s lantern muscle	3	21	−14.5 ± 1.3	7.2 ± 0.7	14.6 ± 0.8	X
Ophiuroidea	*Ophiura* sp.	S	Whole animal without gut	5	23	−17.1 ± 0.9	7.2 ± 0.6	16.5 ± 0.9	X
Holothuroidea	*Heterocucumis* sp.	S	Body wall		23	−25.5 ± 1.0	6.3 ± 1.2	15.9 ± 0.8	X
	*Staurocucumis* sp.	S	Body wall		19	−24.3 ± 0.9	6.4 ± 1.1	16.2 ± 0.7	X

For each item, the table gives the sampling method (S: SCUBA diving, N: Niskin bottle, T: baited traps, H: hand collection), the analysed tissue, the number of specimens sampled in the austral summers of 2013–14 and 2014–15, the stable isotope ratios of carbon (δ^13^C, mean** ± **SD), nitrogen (δ^15^N, mean** ± **SD) and sulfur (δ^34^S, mean** ± **SD) in specimens sampled in 2014–15 and whether the taxon was used for SIAR modelling (in which case there is a “X” in the column “SIAR”).

Most food items had mean δ^15^N ranging from 3 to 6.5‰ (Table 1), with the exception of *H*. *grandifolius* holdfasts (δ^15^N = 9.6 ± 1.2‰) and *P*. *adeliae* guano (δ^15^N = 22.0 ± 1.4‰). *H*. *grandifolius* tissues showed δ^15^N differences (Table 1). However, they were not consistent with δ^13^C variation, as holdfasts were more ^15^N-enriched than stipes (δ^15^N = 3.6 ± 1.3‰; Dunn’s post-hoc test, p < 0.0001) and blades (δ^15^N = 3.2 ± 0.4‰; Dunn’s post-hoc test, p < 0.0001). Mean consumer δ^15^N ranged from 4.4 ± 0.4‰ for the bivalve *Laternula elliptica* to 10.7 ± 0.8‰ for the sea anemone *Isotealia antarctica*. Inside this interval, values were widely overlapping (Table 1).

Sympagic algae had the lowest δ^34^S of food sources (5.6 ± 2.7‰; Table 1), followed by benthic biofilm (10.5 ± 3.0‰). Like for carbon, sulfur isotopic ratios of *H*. *grandifolius* blades (δ^34^S = 17.6 ± 2.7‰) differed from those of stipes (δ^34^S = 16.1 ± 1.0‰; Dunn’s post-hoc test, p = 0.0035) and holdfasts (δ^34^S = 13.1 ± 3.3‰; Dunn’s post-hoc test, p < 0.0001). Finally, SPOM had the highest measured δ^34^S, with values of 18.5 ± 0.8‰. Mean δ^34^S of the vast majority of consumers was mostly overlapping in an interval ranging from 13.5 to 17.5‰ (Table 1). Only four taxa were more ^34^S-depleted, i.e. the sipunculid *Golfingia* sp. (δ^34^S = 12.7 ± 0.7‰), the sea anemone *Isotealia antarctica* (δ^34^S = 11.7 ± 1.5‰), the sponge *Hemigellius* sp. (δ^34^S = 10.9 ± 2.0‰) and the polychaete *Flabegraviera mundata* (δ^34^S = 9.5 ± 2.1‰).

### Resource use by consumers - SIAR mixing model (Fig. 1)

**Figure 1:**
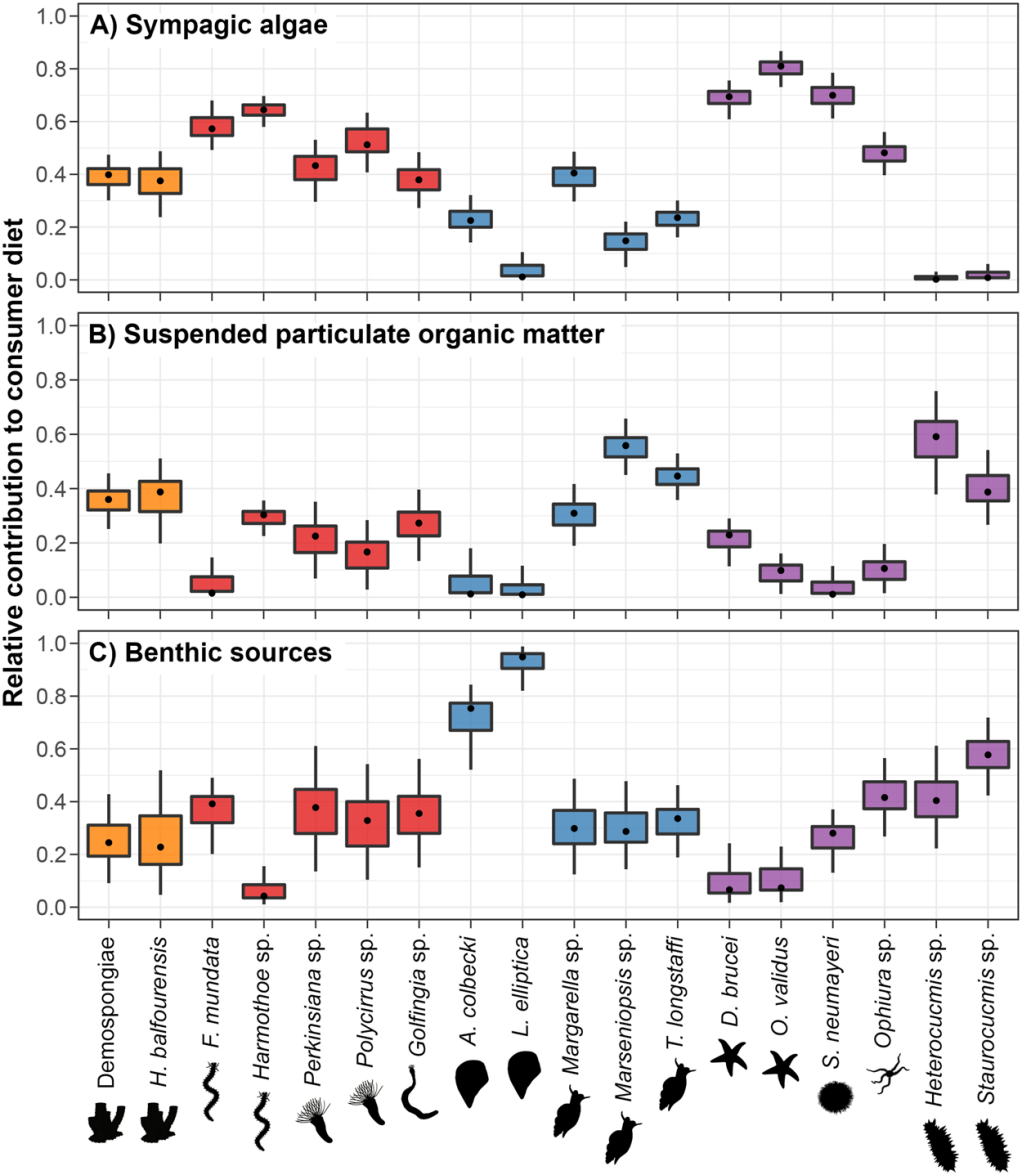
Boxplots of relative contributions of (**A**) sympagic algae, (**B**) suspended particulate organic matter, (**C**) pooled benthic sources (sum of contributions of benthic biofilm and *Himantothallus grandifolius* blades) to invertebrates’ diet, computed using the SIAR model. Orange: Porifera; red: Polychaeta and Sipunculida; blue: Mollusca; purple: Echinodermata. Black dots, boxes and error bars are respectively the modes, 50% and 95% credibility intervals of posterior probability distributions of model solutions.

SIAR outputs (Fig. 1) indicated that sympagic algae were the main food item of 8 of the 18 investigated taxa: polychaetes *Flabegraviera mundata*, *Harmothoe* sp., *Perkinsiana* sp. and *Polycirrus* sp.; sea stars *Odontaster validus* and *Diplasterias brucei*; the sea urchin *Sterechinus neumayeri*; and the brittle star *Ophiura* sp. Contribution of sympagic algae was the highest for *Odontaster validus*, whose 95% credibility interval (CI_95_) ranged from 0.73 to 0.87 with a mode of 0.81 (Fig. 1). Generally speaking, dominance of sympagic algae in the diet was more marked in echinoderms than in polychaetes, where contributions could be moderate (e.g. mode = 0.42 and CI_95_ = [0.30, 0.54] for *Perkinsiana* sp.; Fig. 1).

Four taxa relied on sympagic algae and suspended particulate organic matter (SPOM) in similar proportions: two sponges (*Homaxinella balfourensis* and an unidentified species of Demospongiae), the sipunculid *Golfingia* sp. and the gastropod *Margarella* sp. (Fig. 1). Model outputs suggested that the main resource supporting the gastropods *Marseniopsis* sp. and *Trophonella longstaffi* was SPOM (Fig. 1). Diet of both Dendrochirota sea cucumbers (*Heterocucumis* sp. and *Staurocucumis* sp.) was co-dominated by SPOM and benthic sources (Fig. 1). Difference in the feeding habits of these two species were nonetheless visible, as *Heterocucumis* sp. consumed more SPOM (mode = 0.59; CI_95_ = [0.39, 0.77]) than *Staurocucumis* sp. (mode = 0.39; CI_95_ = [0.27, 0.54]) in 95.98% of model solutions. Accordingly, SPOM was *Heterocucumis* sp.’s main food item, while benthic production was more important than SPOM for *Staurocucumis* sp. (Fig. 1). Finally, benthic food items largely dominated the diet of bivalves *Adamussium colbecki* (mode = 0.75; CI_95_ = [0.52, 0.84]) and *Laternula elliptica* (mode = 0.95; CI_95_ = [0.82, 0.99]: Fig. 1).

### Trophic position estimates (Fig. 2)

**Figure 2:**
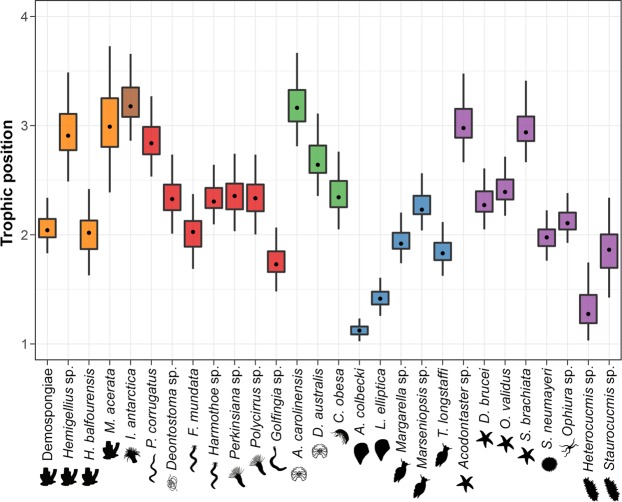
Boxplots of consumers’ trophic positions, estimated using the tRophicPosition model. Orange: Porifera; brown: Cnidaria; red: Nemertea, Nematoda, Polychaeta and Sipunculida; green: Arthropoda; blue: Mollusca; purple: Echinodermata. Black dots, boxes and error bars are respectively the modes, 50% and 95% credibility intervals of posterior probability distributions of model solutions.

The modal trophic position (Fig. 2) of studied organisms varied between 1.12 for *Adamussium colbecki* (CI_95_ = [1.04, 1.21]) and 3.18 for *Isotealia antarctica* (CI_95_ = [2.91, 3.58]). Many invertebrates had low trophic positions (TP), as eight taxa had a modal trophic level inferior to 2 (*Adamussium colbecki*, *Heterocucumis* sp., *Laternula elliptica*, *Golfingia* sp., *Trophonella longstaffi*, *Staurocucumis* sp., *Margarella* sp. and *Sterechinus neumayeri*; Fig. 2). Only six taxa had a modal trophic position close to or higher than 3 (*Hemigellius* sp., *Saliasterias brachiata*, *Acodontaster* sp., *Mycale acerata*, *Ammothea carolinensis* and *Isotealia antarctica*; Fig. 2). Trophic position of the remaining taxa oscillated between 2 and 3. Striking intra-group differences were found in sponges (Fig. 2), as trophic positions of *Hemigellius* sp. (modal TP = 2.91; CI_95_ = [2.56, 3.38]) and *Mycale acerata* (modal TP = 2.99; CI_95_ = [2.50, 3.65]) were higher than those of *Homaxinella balfourensis* (modal TP = 2.02; CI_95_ = [1.69, 2.35]) and the unidentified species of Demospongiae (modal TP = 2.04; CI_95_ = [1.86, 2.29]) in more than 95% of model solutions.

### Inter-annual comparison (Fig. 3)

**Figure 3:**
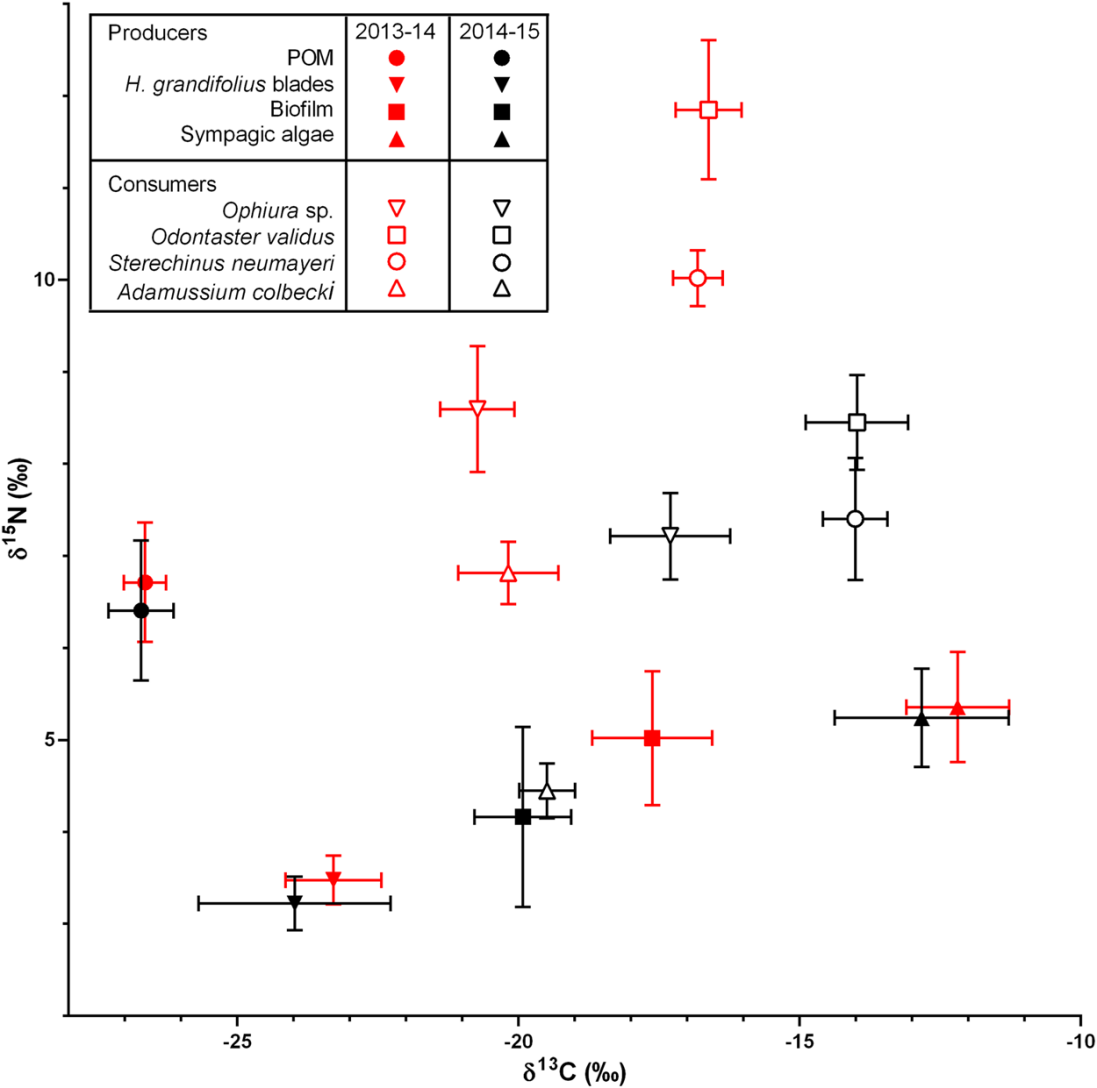
Comparison of stable isotope ratios of carbon and nitrogen of producers/organic matter pools (full symbols) and consumers (empty symbols) sampled during 2013–2014 (red) and 2014–2015 (black) austral summers at site 1 (Anse du Lion). Symbols are means, error bars are standard deviations.

At site 1 (Anse du Lion), the isotopic composition of suspended particulate organic matter, *Himantothallus grandifolius* blades and sympagic algae was similar for both carbon and nitrogen between austral summers 2013–2014 and 2014–2015 (Mann-Whitney U test, p > 0.05 in each case; Fig. 3). Biofilm δ^15^N did not change over time (Mann-Whitney U test, p = 0.0837; Fig. 3), but its δ^13^C showed significant variation (Mann-Whitney U test, p = 0.0012), shifting from −17.6 ± 1.1‰ in 2013–2014 to −19.9 ± 0.9‰ in 2014–2015 (Fig. 3).

Contrastingly, the carbon and nitrogen isotopic ratios of all consumers that could be sampled at site 1 in both years varied strongly. Both δ^13^C and δ^15^N of *Sterechinus neumayeri*, *Odontaster validus* and *Ophiura* sp. significantly shifted over time (Mann-Whitney U test, p < 0.05 in each case; Fig. 3). Most of these changes were striking, sometimes as much as 3‰. Although magnitude of changes was variable, all taxa showed a δ^15^N decrease and a δ^13^C increase (Fig. 3). *Adamussium colbecki* also exhibited considerable δ^15^N decrease (6.8 ± 0.3‰ in 2013–2014 vs. 4.4 ± 0.3‰ in 2014–2015; Mann-Whitney U test, p = 0.0013; Fig. 3), but its δ^13^C remained constant over time (Mann-Whitney U test, p = 0.1909; Fig. 3).

## Discussion

Most of the complexity of trophic interactions can be captured using two fundamental dimensions, leading to the traditional depiction of food webs as bi-dimensional diagrams^18^. Here, we used state-of-the-art models based on well-established trophic tracers (i.e. stable isotope ratios of light biogenic elements) as proxies of both of these food web dimensions. Use of a mixing model allowed us to quantify the relative importance of primary producers for animal nutrition at our study site, and therefore to estimate the diversity of producers sustaining the food web. The trophic position model, on the other hand, provided information on the vertical structure of the food web. Our results suggest that, in zones that typically undergo a sea ice breakup during austral summer (hereafter referred to as “normal conditions”), year-long persistence of sea ice is likely to influence both food web dimensions, as reliance of consumers on sympagic algae seemed higher (change in horizontal structure), while many consumers seemed to occupy lower trophic positions (change in vertical structure) than in normal conditions.

Co-occurrence of three groups of producers, each associated with an ecosystem compartment (i.e. sympagic, pelagic and benthic producers) is a ubiquitous feature of coastal marine systems of Antarctica^22^. Here, mixing model outputs (Fig. 1) pointed out that sympagic algae were very important for the food web, as they dominated or co-dominated the diet of 12 out of 18 investigated taxa. In comparison, pelagic food items dominated or co-dominated the diet of 8 taxa, and benthic producers (macroalgae and biofilm), despite the seemingly huge available biomass, were among the main food items of only 4 out of 18 taxa. This widespread reliance of benthic consumers on sympagic algae strikingly spanned multiple feeding guilds (suspension and deposit feeders, grazers, browsers, and omnivores) and seemed to extend to sessile taxa such as sedentary polychaetes or sponges. During sampling dives, large (several cm in length) algal filaments were observed at the interface between ice and seawater (Supplementary Information S1). Algal aggregates are known to sink quickly upon detachment^23^, and can likely reach the water bottom of shallow zones such as our study areas in a few hours. Aggregates can therefore be available for consumption by benthic animals directly on the substrate, but also during their sinking, or as re-suspended material. Preferential consumption of sympagic algae might be explained by their higher nutritional value and/or palatability. In the Arctic, ice algae are readily consumed by benthic taxa, and may be preferentially selected by some species, possibly due to their high essential fatty acid content^24^. Here, the ratio between elemental contents of carbon and nitrogen (C/N ratio), a commonly used proxy for nutritional quality of a food item, was very high for *Himantothallus grandifolius* blades (14.0 ± 2.2; mean ± SD), which might limit suitability of this food item for some consumers. Moreover, *H*. *grandifolius* has thick and robust blades, and large Phaeophyceae commonly contain high amounts of poorly digestible structural compounds^25^, as well as herbivore deterrents^26^. They might therefore be avoided by some of the studied fauna. C/N ratios were comparable for biofilm (6.2 ± 0.6; mean ± SD) and sympagic algae (6.7 ± 1.2; mean ± SD), suggesting similar nutritional value for both food items. Preferential consumption of sympagic algae could therefore be explained by palatability differences. Microscopic examination of benthic biofilm showed that it was a highly heterogeneous mix of dead and living microalgae, prokaryotes, organic detritus from various origins, and even inorganic material. Some of these elements could act as deterrents for invertebrate consumers, which might instead feed on sea-ice derived material that is likely mostly present in the form of pure sympagic algae aggregates.

Regardless of factors driving it, this predominance of sympagic algae in benthic invertebrate diet does not seem to be a common feature of coastal Antarctic food webs. In the Antarctic Peninsula, phytoplankton, Phaeophyceae and their associated diatoms have been reported to be the main items fuelling food webs from rocky shores^27^. Similarly, soft-bottom consumers can rely on planktonic production and microphytobenthos and macroalgae^28^, that can notably be consumed under detrital form^29^. In East Antarctica, three major carbon pathways were reported: one based on pelagic POM, one on macroalgae and their epiflora, and a last one on sediment POM, macroalgal detritus and benthic diatoms^30^. Sympagic material was not identified as a dominant food item in any of those pathways. In the Ross Sea, studies based on spatial designs suggested that sea ice extent modulates resource use by benthic consumers^14,16^. Interestingly, feeding patterns of consumers seemed to differ from the ones we observed in this study. In the Ross Sea, sympagic inputs to the diet of other consumers such as *Odontaster validus* and *Sterechinus neumayeri* were high but did not vary according to sea ice extent or persistence^16^. Here, stable isotope ratios of both taxa were significantly different between the two sampling years, suggesting that they shifted towards a more sympagic-based diet between the first and the second summer without seasonal sea ice breakup (Fig. 3). Past studies showed that predominance of sediment-associated detritus in the diet of benthic consumers, including the sea urchin *Sterechinus neumayeri*, increased in sea ice-influenced localities^14^. Contrastingly, our mixing model suggests that in Adélie Land, this species relied mostly on sympagic-derived material, and that importance of benthic food items in its diet was limited (Fig. 1). On the other hand, past research pointed out that contribution of sympagic material to diet of *Laternula elliptica* increased in locations with higher sea ice cover^16^. This was not seen here, as our model suggested that this species was one of the few ones to rely quasi-exclusively on benthic production (Fig. 1). These discrepancies might be linked with the peculiar physiology of Antarctic benthic invertebrates. Many consumers studied here have long life spans (sometimes several decades) and low growth and metabolic rates^31–33^. Experimental estimates of isotopic turnover of tissues does not, to the best of our knowledge, exist for Antarctic benthic invertebrates. It has nevertheless been suggested that this turnover time is likely to be high, and could approach a year or more^16,34^. In this context, some of the sampled consumers might not have reached isotopic equilibrium with their food items yet. This would suggest caution when interpreting model outputs, as isotopic equilibrium between consumers and food items is one of the main assumptions underlying them. Consumers could also have a greater selectivity than what was modeled here (e.g. by selectively feeding on specific items among the biofilm). The mixing model built here represents a simplification of reality, and would not be able to detect such fine-scale processes. Nevertheless, the clear changes in isotopic ratios of dominant consumers between the first and the second year without seasonal breakup combined with the absence of consistent changes in isotopic composition of food items (Fig. 3) strongly suggest that the diet of those species actually shifted over this period. These horizontal changes in food web structure, and important reliance of multiple benthic invertebrate taxa on sympagic production could have functional implications, as they could increase competition for resources, and ultimately impact community structure. They could also have biogeochemical implications, as organic matter fluxes between the two situations could differ drastically. On the other hand, these diet shifts could indicate that dominant benthic species are able to readily adjust to changes in food item availability. This trophic plasticity, that could be an adaptive trait in the context of intense seasonality encountered in coastal Antarctica^17^, could also increase resilience of benthic invertebrate fauna to future environmental changes.

Continuous estimates of trophic position (Fig. 2) are commonly used metrics in food web ecology. They provide simple ways to assess organisms’ functional roles in natural ecosystems and to estimate energy flow through ecological communities, while taking into account complex processes such as omnivory^35–37^. This last point is particularly critical for Antarctic zoobenthos, among which food webs are better described as a “trophic continuum” than as discrete trophic levels^30^. Omnivory appears to be an effective way to cope with the strong spatial and temporal changes in food availability characteristic of Antarctic coastal systems, and many consumers have multiple feeding strategies^14,30,38^. Estimates yielded by our trophic position model (Fig. 2) were overall rather low. Many taxa typically regarded as omnivores (e.g. polychaetes, sea stars *Odontaster validus* and *Diplasterias brucei*, the sea urchin *Sterechinus* neumayeri) had a trophic level inferior to 2.5, suggesting that from a functional point of view, they acted mostly as herbivores. Only a few taxa had trophic positions over 3, suggesting they were actually secondary consumers. To the best of our knowledge, none of the studied taxa significantly rely on mixotrophy. It is therefore likely that animals showing TP inferior to 2 (e.g. *Adamussium colbecki*, *Laternula elliptica*, *Heterocucumis* sp.) were either not at isotopic equilibrium with their food sources, or selectively feeding on low δ^15^N items. Nevertheless, δ^15^N of some abundant consumers such as *S*. *neumayeri* and *O*. *validus* showed a significant decrease between the two sampling years, while the δ^15^N of food items remained constant over the same period of time (Fig. 3). This demonstrates that the trophic position of some taxa decreased between the first and second year without seasonal breakup. Contrastingly, previous research lead in East Antarctica suggested that many consumers (including *O*. *validus*) had trophic levels markedly superior to 3^30^. Comparable findings were reported from the Antarctic Peninsula^27^. Moreover, in the Ross Sea, δ^15^N of benthic invertebrates tended to be higher, and animal-derived matter contributed more to the diet of some taxa (e.g. *S*. *neumayeri*) in stations with high sea ice influence, presumably because of lower macrophyte productivity and diversity^14^.

Here, unusual absence of seasonal breakup could have caused a decline in animal prey availability for benthic consumers. While faunal densities were not quantified in our study, occurrence of taxa reported as abundant during previous investigations at the same site seemed moderate, forcing us to increase sampling effort to reach adequate replication. This is in agreement with other observations from East Antarctica, where, although Ophiuroidea seemed able to cope with such conditions, anomalous year-round sea ice persistence was linked with low overall abundance of fauna^13^. Low trophic positions of some consumers, including biomass-dominant and ecologically important omnivores, could be another hint at high trophic plasticity among Antarctic zoobenthos. They could indicate that, when faced with high availability of suitable plant-derived material, animals are able to shift their diet to take advantage of this food supply. Important availability of basal resources has accordingly been associated with a decrease of trophic level in predator^39^ as well as Arctic omnivore invertebrates^40^. Low trophic positions nevertheless raise concerns about energy flow among benthic communities in the absence of sea-ice breakup. Each animal consumer indeed needs sufficient energy to thrive, and has to cope with stoechiometrical constraints^41^. Some of the taxa showing surprisingly low trophic positions here might have trouble meeting their energy and/or nutrient demands. This could have adverse effects on secondary production and population dynamics, and, ultimately, community structure. In this context, alternative feeding strategies might be an efficient way to deal with nutritional stress. Here, strong intra-group differences in trophic positions were pointed out in sponges (Fig. 2). Sponges harbor a variety of symbionts capable of heterotrophic and autotrophic metabolism^42^, which can represent up to 35% of sponge biomass^43^. In abyssal plains, it has been suggested that symbiotic bacteria could be able to metabolize refractory material, which can then be assimilated by the sponge. This process leads to an elevation of δ^15^N of sponges^44^. Something similar could happen here, and the widely different trophic levels found in sponges could reflect different levels of reliance on symbiont-mediated trophic processes.

Patterns of food web structure under increased sea ice conditions in Adélie Land seem different from those suggested by previous studies from the Ross Sea^14,16^. These discrepancies might be driven by local variation in environmental parameters such as sea ice thickness and cover that will in turn influence under-ice light availability. Moreover, both studies report that sea ice conditions across their surveys were consistent over long time periods, suggesting that organisms had time to adapt to environmental constraints and, that communities had time reach stable structure. In our case, however, rapid (when compared to invertebrate life spans) environmental changes could cause instability in community structure and food supply to the benthos. This could explain the clear changes in feeding habits of dominant consumers between the first and second year without breakup. Timing, frequency and extent of sea ice breakup could therefore be essential parameters to consider to achieve a satisfactory understanding of how current changes in sea ice cover influence benthic food webs in Antarctica.

## Conclusions

When compared with food webs described in contrasting but stable sea ice conditions, our results suggest that the absence of seasonal breakup causes simplification of the food web in both its fundamental dimensions. Horizontal trophic interactions, indicating food/energy sources, were characterized by low diversity of food items supporting animal population and high reliance on sympagic material. Vertical food web structure seemed weakly defined, with many consumers occupying low and overlapping trophic positions. In East Antarctica, zoobenthos richness and abundance is positively correlated with food availability^45^. Food web modifications could therefore have implications for ecosystem functioning and, ultimately, community structure. Holistic, quantitative approaches attempting to link environmental changes to the role of species in ecological networks have accordingly been suggested as an efficient way to understand impacts of global change on Antarctic ecosystems^46^. In addition to the processes we document here, some impressive ecosystem changes, such as unprecedented development of biofilm that overgrows macroalgae and sessile consumers (Supplementary Information S1), or discoloration of macroalgae, were observed but not quantified. These changes are akin those described in other parts of Antarctica, where abnormal year-round persistence of sea ice in relation to iceberg calving strongly impacted benthic communities, possibly triggering an ecosystem phase shift^13^. Results presented here suggest that changes in food supply and trophic interactions could be one of the mechanisms through which this phase shift could take place.

When faced with such substantial environmental changes, animals can either adapt, migrate or disappear^47^. Migration capabilities of Antarctic zoobenthos are often limited due to low mobility and/or absence of dispersing larval stages^48^. On the other hand, our results support the hypothesis that absence of seasonal sea ice breakup caused benthic invertebrates to shift their diet. At the seasonal timescale, trophic plasticity of coastal Antarctic zoobenthos is regarded as beneficial^17^. It could allow organisms to take advantage of the short-term shifts in resource availability, which translates to an increase in metabolic activity^49^, in nutritional status and energy content^50^, and in reproductive activity^51^. In the longer term, trophic plasticity of Antarctic invertebrates could mediate adaptation to future environmental changes. Integrative studies attempting to relate dietary changes to individual and/or population-level assessments of fitness would provide valuable insights regarding how taxa cope with to their new environmental constraints. Consumer identity will likely modulate response to environmental changes, as feeding habits could show important variation among some studied taxonomical groups (e.g. sponges, bivalves, pycnogonids, sea stars and sea cucumbers; Figs 1 and 2). Similarly, the shift in isotopic values between the two sampled years differed between some consumers in both magnitude and direction (Fig. 3).

More research is needed to understand how the magnitude of environmental changes (e.g. varying levels of sea ice thickness and/or snow cover) could further modulate food web architecture. How community resilience could help benthic ecosystems to recover from such extreme sea ice events also remains an open question. However, many ecological processes are slow in Antarctica^52^, while the recent frequency of austral summers without sea ice breakup in coastal Adélie Land has been high, with normal breakup conditions happening only twice over the past 5 years. In this context, local or global trends of temporal or spatial increase of sea ice cover might have strong impacts on benthic invertebrate communities.

## Methods

### Sampling

Sampling took place in the surroundings of Dumont-d’Urville station (French Polar Institute Paul-Emile Victor - IPEV), on Petrels Island (Adélie Land, East Antarctica) during the austral summers of 2013–2014 (22–26/01/2014) and 2014–2015 (17/12/2014–12/01/2015). During the sampling period, fast ice did not undergo seasonal breakup for two successive years (ice thickness fluctuating between 40 and over 200 cm during summer, cf. Supplementary Information S2). Snowfall was negligible (i.e. too low to be measured) throughout all the period (Supplementary Information S2). Two sampling sites were chosen. Site 1 (“Anse du Lion”; 140.003°E, 66.661°S) was visited during both campaigns, while site 2 (“Cap des Eléphants”; 139.997°E, 66.667°S) was sampled only in 2014–2015. In both sampling campaigns, both sites were covered by a thick layer of fast ice (from around 100 to over 200 cm; Supplementary Information S2), and holes were drilled to allow access to the sea.

In total, 28 invertebrate taxa (Table 1) spanning 9 phyla and most common functional guilds in coastal Antarctica were sampled at depths ranging from 10 to 20 m. Most invertebrate samples were collected by SCUBA divers, either by hand or using small landing nets, but some specimens were caught using small traps baited with fish tissues (Table 1).

Six items (producers or organic matter pools, Table 1) were identified as potential food sources for primary consumers. Sympagic algae, which mostly occurred as several cm long filaments (Supplementary Information S1), were sampled by SCUBA divers by scraping the lower surface of fast ice. The dominant macroalgae, the large Phaeophyceae *Himantothallus grandifolius* and the Rhodophyceae *Phyllophora antarctica*, were hand-collected by SCUBA divers. Benthic biofilm was collected by scraping rocks *in situ*. It was scarce in 2013–2014 but extremely abundant in 2014–2015 (thick layer of several cm covering rocks but also macroalgae and sponges, Supplementary Information S1). Seawater for suspended particulate organic matter (SPOM) was collected through the diving holes, at 10 m depth, using a Niskin bottle. Seawater was then pre-sieved to remove items larger than 5 mm, and filtered on pre-combusted (4 h at 400 °C) glass fibre filters (Whatman GF/F, sieve size 0.7 µm). For each SPOM sample, 20 litres of seawater were filtered. Finally, samples of the abundant deposits of guano surrounding the extensive colonies of Adélie penguins (*Pygoscelis adeliae*) were hand collected on land, in the vicinity of the diving holes.

### Stable isotope analysis

At Dumont-d’Urville station, animal samples were placed in aerated seawater tanks immediately after collection and processed as soon as possible. Animals were dissected to separate soft and non-metabolically active tissues^53^. Due to important differences in invertebrate body structure and size, selected tissues were not the same for each taxon (Table 1). Most food items were processed whole, with the exception of *Himantothallus grandifolius* for which holdfasts, stipes and blades were separated. All samples were oven-dried at 60 °C for 72 h, then placed in airtight containers and kept at room temperature before further treatment once back from the expedition. They were subsequently ground to a homogeneous powder using mortar and pestle or, when required, a MM301 mixer mill (Retsch GmBH, Haan, Germany) (cycles of 60 seconds at 25 Hz).

Inorganic carbon present in samples can be a source of bias in carbon stable isotope analysis. “Champagne tests” where therefore used to highlight presence of carbonates in tissues^54^. They revealed that tissues of *Margarella* sp., *Acodontaster* sp., *Diplasterias brucei*, *Odontaster validus*, *Saliasterias brachiata*, *Sterechinus neumayeri*, *Ophiura* sp., *Heterocucumis* sp. and *Staurocucumis* sp. contained moderate yet significant amounts of carbonates. They were therefore acidified by exposing them to HCl vapours for 48 h in an airtight container^55^. After acidification, a second series of “champagne tests” were run. They indicated that the procedure successfully removed all carbonates from samples. Since acidification can alter N^53^ and S^56^ isotopic ratios, acidified samples were analysed twice: once for C isotopic ratios, using decarbonated material, and once for N and S isotopic ratios, using native material.

Stable isotope ratios measurements were performed via continuous flow - elemental analysis - isotope ratio mass spectrometry (CF-EA-IRMS) at University of Liège (Belgium), using a vario MICRO cube C-N-S elemental analyser (Elementar Analysensysteme GMBH, Hanau, Germany) coupled to an IsoPrime100 isotope ratio mass spectrometer (Isoprime, Cheadle, United Kingdom). Isotopic ratios were expressed using the widespread δ notation^57^, in ‰ and relative to the international references Vienna Pee Dee Belemnite (for carbon), Atmospheric Air (for nitrogen) and Vienna Canyon Diablo Troilite (for sulfur). IAEA (International Atomic Energy Agency, Vienna, Austria) certified reference materials sucrose (IAEA-C-6; δ^13^C = −10.8 ± 0.5‰; mean ± SD), ammonium sulphate (IAEA-N-2; δ^15^N = 20.3 ± 0.2‰; mean ± SD) and silver sulfide (IAEA-S-1; δ^34^S = −0.3‰) were used as primary analytical standards. Sulfanilic acid (Sigma-Aldrich; δ^13^C = −25.6 ± 0.4‰; δ^15^N = −0.13 ± 0.4‰; δ^34^S = 5.9 ± 0.5‰; means ± SD) was used as secondary analytical standard. Standard deviations on multi-batch replicate measurements of secondary and internal lab standards (amphipod crustacean muscle) analysed interspersed with samples (one replicate of each standard every 15 analyses) were 0.2‰ for both δ^13^C and δ^15^N and 0.5‰ for δ^34^S.

### Data treatment

Unless noted otherwise, all values are presented as mean ± SD. Differences in δ^13^C, δ^15^N and δ^34^S were tested using hypothesis-based comparison procedures. D′Agostino & Pearson and Shapiro-Wilk normality tests revealed that several datasets did not follow a Gaussian distribution. Non-parametric procedures (Mann-Whitney U test when 2 groups were compared, Kruskal-Wallis one-way analysis of variance followed by Dunn’s post-hoc test when 3 groups or more were compared) were therefore applied. All statistical analyses were conducted using Prism 6.05 (GraphPad Software, La Jolla, U.S.A.). In the vast majority of cases, no inter-site difference in δ^13^C, δ^15^N or δ^34^S of consumers or food items was found, motivating our decision to pool all measurements made in 2014–2015 (Supplementary Information S3; Table 1).

To quantify resource use by primary consumers and omnivores, we used the Bayesian SIAR (Stable Isotope Analysis in R) mixing model^20^. Models were built only for 2014–2015 data, as replication for the 2013–2014 samples was low (Table 1). Four food sources were held for modelling purposes: suspended particulate organic matter, benthic biofilm, sympagic algae and *Himantothallus grandifolius* blades. *Phyllophora antarctica* blades and *Pygoscelis adeliae* guano were discarded because of their extreme δ^13^C and δ^15^N values, respectively (see “Results” section for details). *Himantothallus grandifolius* stipes and holdfasts were discarded because their tough tissues likely renders them unpalatable for most marine invertebrates. Primary consumers and omnivores were selected by analysing their δ^15^N values (see “Results” section for more details). Considering the highest mean δ^15^N found in food items (suspended particulate organic matter; δ^15^N = 6.71‰) and a mean N trophic enrichment factor (TEF) of 2.30‰^58^, we considered that organisms were primary consumers or omnivores when their δ^15^N was less than 2.30‰ over suspended particulate organic matter, i.e. equal or inferior to 8.71‰. It was the case for 18 invertebrate taxa (Table 1). Since there are no specific TEFs for the taxa studied here, we used widely applicable values, i.e. 0.4 ± 1.2‰ for C, 2.3 ± 1.6‰ for N and 0.5 ± 1.9‰ for S (mean ± SD in each case)^58^. Models were run using the SIAR 4.2 package in R 3.3.2^59^. Iteration numbers and burn-in size were set at 10^6^ and 10^5^, respectively. Two modelling scenarios were considered: one using δ^13^C, δ^15^N and δ^34^S, and the other using only δ^13^C and δ^15^N. Model diagnostics surprisingly indicated that the scenario using all three isotopic ratios showed poorer performance. This might be caused by a combination of factors: 1) δ^34^S values of food items showed a high variability (Table 1); 2) δ^34^S values of sympagic algae and biofilm, as well as those of *H*. *grandifolius* blades and SPOM were statistically identical (Dunn’s post-hoc test, p = 0.2387 and p > 0.9999, respectively), leading to poor discrimination of those sources by the model; and 3) experimental measurements of sulfur trophic fractionation in aquatic consumers are extremely scarce, which forced us to use a supposedly generally applicable TEF that might not be fully suitable.

The scenario using only δ^13^C and δ^15^N was retained and presented here. Model solutions were presented using credibility intervals of probability density function distributions^20^. Benthic biofilm and *H*. *grandifolius* were treated as different food items for modelling. However, in order to have a proxy of total benthic production inputs to invertebrate diet, their contributions were summed a posteriori and referred to as “benthic sources”. When relevant, direct pairwise comparisons of model-estimated contributions were performed. Those comparisons were considered meaningful when probability of occurrence exceeded 95%.

To estimate trophic position (TP) of invertebrates, we used the Bayesian model tRophicPosition 0.5.0.1000 20 in R 3.3.2^21^. Trophic position estimates were performed only for 2014–2015 data, as replication for 2013–2014 samples was low (Table 1). Models were run using δ^13^C and δ^15^N values of consumers, TEFs of 0.4 ± 1.2‰ for C and 2.3 ± 1.6‰ for N, and taking into account two baseline items directly^60^. For taxa for which SIAR modelling had been performed, model output was used to select the two most relevant baseline items (i.e. the two items contributing the most to animal diet). For others, sympagic algae and suspended particulate organic matter were used as baseline items. Trophic position of basal food items was set to 1.0, meaning that a TP of 2.0 represents a primary consumer, 3.0 a secondary consumer, etc. For each taxon, two parallel chains were sampled with 10000 adaptive iterations. Model solutions were presented using credibility intervals of probability density function distributions. When relevant, direct pairwise comparisons of model-estimated trophic positions were performed. Those comparisons were considered meaningful when probability of occurrence exceeded 95%.

## Supplementary information

Supplementary info S1-S3

Supplementary information S1 – Video footage of sampling conditions

## Data Availability

All data supporting this article are openly available via the Antarctic Biodiversity Information Facility (ANTABIF, www.biodiversity.aq) at https://ipt.biodiversity.aq/resource?r=ddu_isotopes_verso_2013_2015. They are also registered at the Global Biodiversity Information Facility (GBIF) under UUID 90f2713a-79ac-4d96–9a66-889a5fb9abb1, and freely accessible at 10.15468/wgfw0h.

## References

[1] Barnes DKA, Peck LS (2008). Vulnerability of Antarctic shelf biodiversity to predicted regional warming. Clim. Res..

[2] Portner HO, Peck L, Somero G (2007). Thermal limits and adaptation in marine Antarctic ectotherms: an integrative view. Philos. Trans. R. Soc. B Biol. Sci..

[3] Parkinson CL, Cavalieri DJ (2012). Antarctic sea ice variability and trends, 1979-2010. Cryosphere.

[4] Turner J (2005). Antarctic climate change during the last 50 years. Int. J. Climatol..

[5] Massom RA, Stammerjohn SE (2010). Antarctic sea ice change and variability - Physical and ecological implications. Polar Sci..

[6] King JA (2014). Resolution of the Antarctic paradox. Nature.

[7] Turner J, Harangozo SA, Marshall GJ, King JC, Colwell SR (2002). Anomalous atmospheric circulation over the Weddell Sea, Antarctica during the Austral summer of 2001/02 resulting in extreme sea ice conditions. Geophys. Res. Lett..

[8] Fraser AD, Massom RA, Michael KJ, Galton-Fenzi BK, Lieser JL (2012). East antarctic landfast sea ice distribution and variability, 2000-08. J. Clim..

[9] Fripiat F, Sigman DM, Massé G, Tison JL (2015). High turnover rates indicated by changes in the fixed N forms and their stable isotopes in Antarctic landfast sea ice. J. Geophys. Res. Ocean..

[10] Massom RA (2009). Fast ice distribution in Adélie Land, East Antarctica: Interannual variability and implications for emperor penguins *Aptenodytes forsteri*. Mar. Ecol. Prog. Ser..

[11] Barbraud, C. & Weimerskirch, H. Antarctic birds breed later in response to climate change. *Proc*. *Natl*. *Acad*. *Sci*. **103**, 6248–6251 (2006). 10.1073/pnas.0510397103 PMC1458863 16601100

[12] Kusahara K (2017). Modeling Ocean–Cryosphere Interactions off Adélie and George V Land, East Antarctica. J. Clim..

[13] Clark GF, Marzinelli EM, Fogwill CJ, Turney CSM, Johnston EL (2015). Effects of sea-ice cover on marine benthic communities: a natural experiment in Commonwealth Bay, East Antarctica. Polar Biol..

[14] Norkko A (2007). Trophic structure of coastal Antarctic food webs associated with changes in sea ice and food supply. Ecology.

[15] Michel LN, David B, Dubois P, Lepoint G, De Ridder C (2016). Trophic plasticity of Antarctic echinoids under contrasted environmental conditions. Polar Biol..

[16] Wing SR, McLeod RJ, Leichter JJ, Frew RD, Lamare MD (2012). Sea ice microbial production supports Ross Sea benthic communities: Influence of a small but stable subsidy. Ecology.

[17] Calizza E, Careddu G, Sporta Caputi S, Rossi L, Costantini ML (2018). Time- and depth-wise trophic niche shifts in Antarctic benthos. PLoS One.

[18] Cagnacci Francesca, Boitani Luigi, Powell Roger A., Boyce Mark S. (2010). Animal ecology meets GPS-based radiotelemetry: a perfect storm of opportunities and challenges. Philosophical Transactions of the Royal Society B: Biological Sciences.

[19] Parnell AC (2013). Bayesian stable isotope mixing models. Environmetrics.

[20] Parnell AC, Inger R, Bearhop S, Jackson AL (2010). Source partitioning using stable isotopes: Coping with too much variation. PLoS One.

[21] Quezada-Romegialli C (2018). tRophicPosition, an R package for the Bayesian estimation of trophic position from consumer stable isotope ratios. Methods Ecol. Evol..

[22] Knox G. A. (1990). Primary Production and Consumption in McMurdo Sound, Antarctica. Antarctic Ecosystems.

[23] Riebesell U, Schloss I, Smetacek V (1991). Aggregation of algae released from melting sea ice: implications for seeding and sedimentation. Polar Biol..

[24] McMahon KW (2006). Benthic community response to ice algae and phytoplankton in Ny Ålesund, Svalbard. Mar. Ecol. Prog. Ser..

[25] Bruhn A (2017). Crude fucoidan content in two North Atlantic kelp species, *Saccharina latissima* and *Laminaria digitata*—seasonal variation and impact of environmental factors. J. Appl. Phycol..

[26] Duggins DO, Eckman JE (1997). Is kelp detritus a good food for suspension feeders? Effects of kelp species, age and secondary metabolites. Mar. Biol..

[27] Dunton KH (2001). δ^15^N and δ^13^C Measurements of Antarctic Peninsula Fauna: Trophic Relationships and Assimilation of Benthic Seaweeds. Am. Zool..

[28] Corbisier TN, Petti MAV, Skowronski RSP, Brito TAS (2004). Trophic relationships in the nearshore zone of Martel Inlet (King George Island, Antarctica): δ^13^C stable-isotope analysis. Polar Biol..

[29] Pasotti F (2015). Benthic trophic interactions in an Antarctic shallow water ecosystem affected by recent glacier retreat. PLoS One.

[30] Gillies CL, Stark JS, Johnstone GJ, Smith SDA (2012). Carbon flow and trophic structure of an Antarctic coastal benthic community as determined by δ^13^C and δ^15^N. Estuar. Coast. Shelf Sci..

[31] Peck LS, Convey P, Barnes DKA (2006). Environmental constraints on life histories in Antarctic ecosystems: Tempos, timings and predictability. Biol. Rev. Camb. Philos. Soc..

[32] Agüera A, Collard M, Jossart Q, Moreau C, Danis B (2015). Parameter estimations of Dynamic Energy Budget (DEB) model over the life history of a key Antarctic species: The Antarctic sea star Odontaster validus Koehler, 1906. PLoS One.

[33] Agüera A, Ahn I-Y, Guillaumot C, Danis B (2017). A Dynamic Energy Budget (DEB) model to describe *Laternula elliptica* (King, 1832) seasonal feeding and metabolism. PLoS One.

[34] Fry, B. *Stable Isotope Ecology*. (Springer, 2006).

[35] Hairston NG, Hairston NG (1993). Cause-Effect Relationships in Energy Flow, Trophic Structure, and Interspecific Interactions. Am. Nat..

[36] Post DM (2002). Using stable isotopes to estimate trophic position: models, methods, and assumptions. Ecology.

[37] Vander Zanden MJ, Rasmussen JB (1999). Primary consumers δ^13^C and δ^15^N and the trophic position of aquatic consumers. Ecology.

[38] Smale DA, Barnes DKA, Fraser KPP, Mann PJ, Brown MP (2007). Scavenging in Antarctica: Intense variation between sites and seasons in shallow benthic necrophagy. J. Exp. Mar. Bio. Ecol..

[39] Calizza E, Costantini ML, Rossi D, Carlino P, Rossi L (2012). Effects of disturbance on an urban river food web. Freshw. Biol..

[40] McMeans BC, McCann KS, Humphries M, Rooney N, Fisk AT (2015). Food Web Structure in Temporally-Forced Ecosystems. Trends Ecol. Evol..

[41] Sterner, R. W. & Elser, J. J. *Ecological Stoichiometry: The Biology of Elements from Molecules to the Biosphere*. (Princeton University Press, 2002).

[42] Webster NS, Thomas T (2016). The Sponge Hologenome. MBio.

[43] Weisz JB, Lindquist N, Martens CS (2008). Do associated microbial abundances impact marine demosponge pumping rates and tissue densities?. Oecologia.

[44] Iken K, Brey T, Wand U, Voigt J, Junghans P (2001). Food web structure of the benthic community at the Porcupine Abyssal Plain (NE Atlantic): A stable isotope analysis. Prog. Oceanogr..

[45] Jansen J (2018). Abundance and richness of key Antarctic seafloor fauna correlates with modelled food availability. Nat. Ecol. Evol..

[46] Ortiz M (2017). Quantifying keystone species complexes: Ecosystem-based conservation management in the King George Island (Antarctic Peninsula). Ecol. Indic..

[47] Williams SE, Shoo LP, Isaac JL, Hoffmann AA, Langham G (2008). Towards an Integrated Framework for Assessing the Vulnerability of Species to Climate Change. PLoS Biol..

[48] Barnes DKA, Conlan KE (2007). Disturbance, colonization and development of Antarctic benthic communities. Philos. Trans. R. Soc. B Biol. Sci..

[49] Brockington S, Peck L (2001). Seasonality of respiration and ammonium excretion in the Antarctic echinoid Sterechinus neumayeri. Mar. Ecol. Prog. Ser..

[50] Brockington S, Clarke A, Chapman ALG (2001). Seasonality of feeding and nutritional status during the austral winter in the Antarctic sea urchin Sterechinus neumayeri. Mar. Biol..

[51] Pearse JS, McClintock JB, Bosch I (1991). Reproduction of Antarctic Benthic Marine Invertebrates: Tempos, Modes, and Timing. Am. Zool..

[52] Stanwell-Smith D, Barnes DK (1997). Benthic community development in Antarctica: recruitment and growth on settlement panels at Signy Island. J. Exp. Mar. Bio. Ecol..

[53] Mateo MA, Serrano O, Serrano L, Michener RH (2008). Effects of sample preparation on stable isotope ratios of carbon and nitrogen in marine invertebrates: implications for food web studies using stable isotopes. Oecologia.

[54] Jaschinski S, Hansen T, Sommer U (2008). Effects of acidification in multiple stable isotope analyses. Limnol. Oceanogr. Methods.

[55] Hedges JI, Stern JH (1984). Carbon and nitrogen determinations of carbonate-containing solids. Limnol. Oceanogr..

[56] Connolly RM, Schlacher TA (2013). Sample acidification significantly alters stable isotope ratios of sulfur in aquatic plants and animals. Mar. Ecol. Prog. Ser..

[57] Coplen TB (2011). Guidelines and recommended terms for expression of stable-isotope-ratio and gas-ratio measurement results. Rapid Commun. Mass Spectrom..

[58] McCutchan JHJ, Lewis WM, Kendall C, McGrath CC (2003). Variation in trophic shift for stable isotope ratios of carbon, nitrogen, and sulfur. Oikos.

[59] R Core Team, N. R: *A Language and Environment for Statistical Computing*. (R Foundation for Statistical Computing, 2016).

[60] Cabana G, Rasmussen JB (1994). Modelling food chain structure and contaminant bioaccumulation using stable nitrogen isotopes. Nature.

