# Supplementary material for: Increased sea ice cover alters food web structure in East Antarctica
Source: Sci Rep. 2019 May 30;9:8062. doi: 10.1038/s41598-019-44605-5 (PMC6542827; doi:10.1038/s41598-019-44605-5)
Supplement: Supplementary file 1 — Supplementary info S1-S3 [file 41598_2019_44605_MOESM1_ESM.pdf]

Increased sea ice cover alters food web structure in East Antarctica – Supplementary information

Loïc N. MICHEL, Bruno DANIS, Philippe DUBOIS, Marc ELEAUME, Jérôme FOURNIER, Cyril GALLUT, Philip JANE & Gilles LEPOINT

Supplementary information S1 – Video footage of sampling conditions

Underwater footage taken during sampling campaign of 2014-15 at site 2 (Cap des Eléphants) and showing the extremely abundant biofilm covering rocks and macroalgae, as well as benthic animals and sympagic algae filaments. In addition to being appended to the manuscript as supplementary material the video can also be seen at https://www.youtube.com/watch?v=fTNziVltIYE.

Supplementary information S2 – Environmental parameters

Ice thickness: Weekly routine measurements were realized, whenever possible, by Dumont-d'Urville station personnel (French Polar Institute Paul-Emile Victor - IPEV) using a handheld ice drill and a measuring tape. The length of the drill was 2 meters, and ice thicknesses superior to that length could therefore not be recorded.

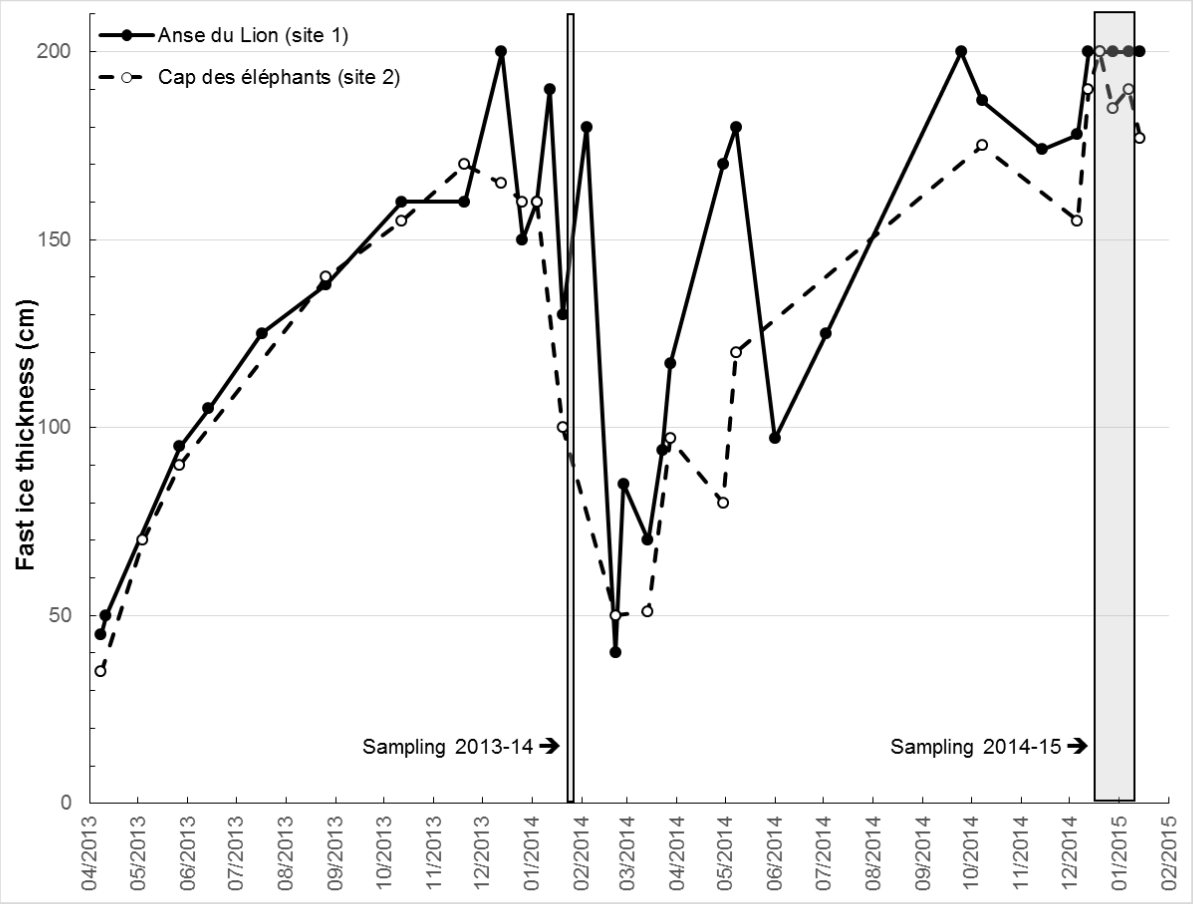

Evolution of fast ice thickness at the two sampling sites from April 2013 to January 2015.

Snowfall: Snowfall occurrence was recorded daily at the Dumont-d'Urville weather station (Météo France / French Polar Institute Paul-Emile Victor - IPEV). Throughout the sampling period, all snowfall events were associated with immediate dispersion of the snow by strong winds, leading to a negligible (i.e. too small to be measured) deposited snow cover.

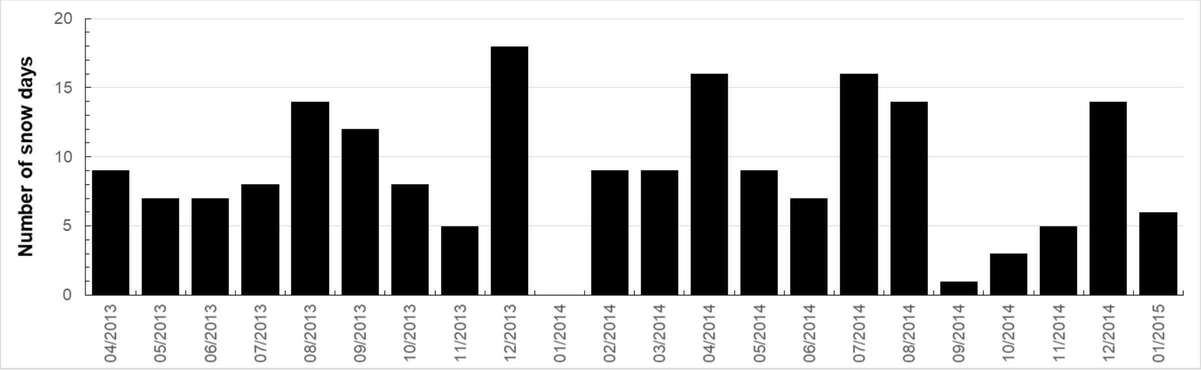

Monthly sums of days with snowfall at Dumont-d'Urville station from April 2013 to January 2015.

Supplementary information S3 – Inter-site comparisons

Summary of inter-site differences in stable isotope ratios of consumers and food items sampled in 2014-2015. For each comparison, the table gives the p-value of the Mann-Whitney test. Green: no difference ($p > 0.05$), red: significant difference ($p < 0.05$). Overall, 80 of 87 comparisons (92%) were non-significant. Only one species showed an inter-site difference in two isotopic ratios, and none showed differences for all three ratios.

	$\delta^{13}C$	$\delta^{15}N$	$\delta^{34}S$
Himantothallus grandifolius - holdfasts	0.6536	0.4732	0.9778
Himantothallus grandifolius - stipes	0.5798	0.4261	0.5777
Himantothallus grandifolius - blades	0.6159	0.8991	0.3734
Phyllophora antarctica	0.7313	0.6745	0.9444
Sympagic algae	0.5409	0.5895	0.8391
Biofilm	0.5134	0.4697	0.0405
Demospongiae Indet.	0.1434	0.4540	0.8322
Hemigellius sp.	0.2326	0.0717	0.0056
Homaxinella balfourensis	0.5279	0.0075	0.1824
Mycale acerata	0.9038	0.1740	0.7251
Parborlasia corrugatus	0.0983	0.1042	0.2189
Deontostoma sp.	0.6848	0.8939	0.9030
Flabegraviera mundata	0.4654	0.0007	0.0055
Harmothoe sp.	0.0260	0.2812	0.0613
Perkinsiana sp.	0.2661	0.1174	0.4266
Polycirrus sp.	0.1741	0.5494	0.1480
Decolopoda australis	0.9093	0.7084	0.2851
Charcotia obesa	0.6106	0.1582	0.6900
Adamussium colbecki	0.9342	0.5857	0.6081
Laternula elliptica	0.5181	0.4803	0.1701
Margarella sp.	0.6169	0.2316	0.8290
Acodontaster sp.	0.6182	0.7606	0.6727
Diplasterias brucei	0.9578	0.2172	0.0993
Odontaster validus	0.9155	0.0812	0.1943
Saliasterias brachiata	0.6783	0.4364	0.7329
Sterechinus neumayeri	0.1977	0.3675	0.9246
Ophiura sp.	0.4955	0.9379	0.3005
Heterocucumis sp.	0.6207	0.5046	0.0012
Staurocucumis sp.	0.5212	0.0615	0.1856
